# GC-MS Screening for the Identification of Potential Migrants Present in Polymeric Coatings of Food Cans

**DOI:** 10.3390/polym11122086

**Published:** 2019-12-13

**Authors:** Antía Lestido Cardama, Raquel Sendón, Juana Bustos, M. Isabel Santillana, Perfecto Paseiro Losada, Ana Rodríguez Bernaldo de Quirós

**Affiliations:** 1 Department of Analytical Chemistry, Nutrition and Food Science, Faculty of Pharmacy, University of Santiago de Compostela, 15782-Santiago de Compostela, Spain; antia.lestido@usc.es (A.L.C.); raquel.sendon@usc.es (R.S.); perfecto.paseiro@usc.es (P.P.L.); 2 National Food Center, Spanish Agency of Food Safety and Nutrition, E-28220 Majadahonda, Spain; JBustos@mscbs.es (J.B.); MSantillana@mscbs.es (M.I.S.)

**Keywords:** screening, GC-MS, LC-MS/MS, BADGE, cans, cyclo-di-BADGE

## Abstract

The coatings used in cans can release complex chemical mixtures into foodstuffs. Therefore, it is important to develop analytical methods for the identification of these potential migrant compounds in packaged food to guarantee the compliance with European food packaging legislation and ensure consumer safety. In the present work, the type of coating in a total of twelve cans collected in Santiago de Compostela (Spain) were evaluated using an ATR (attenuated total reflectance)-FTIR spectrometer. These samples were analysed after extraction with acetonitrile in order to identify potential migrants through a screening method by gas chromatography coupled to mass spectrometry (GC-MS). A total of forty-seven volatile and semi-volatile compounds were identified in these samples, including plasticizers, photoinitiators, antioxidants, lubricants, etc. Then, in a second step, a targeted analysis was carried out for the simultaneous determination of 13 compounds, including bisphenols (BPA, BPB, BPC, BPE, BPF, BPG) and BADGEs (BADGE, BADGE.H_2_O, BADGE.2H_2_O, BADGE.HCl, BADGE.2HCl, BADGE.H_2_O.HCl, cyclo-di-BADGE) by liquid chromatography coupled to tandem mass spectrometry (LC-MS/MS) with atmospheric pressure chemical ionisation (APCI) source. Among all the bisphenols analysed, only the bisphenol A was detected in four samples; while cyclo-di-BADGE was the predominant compound detected in all the samples analysed.

## 1. Introduction

Independently of the material used in the manufacturing (tin, plate, tin-free steel, or aluminium) and the production process, cans for foods and beverages are typically coated on the internal and external side with an organic layer of 1 to 10 µm thickness. This coating is essential for protecting the integrity of the can from the effects of the food, and also to prevent chemical reactions between the can and the food that could lead to food contamination. To comply with technical and legal requirements, can coatings should withstand the production and sterilization processes, prevent chemical migration into food in quantities that endanger human health, adhere to the can even after non-intentional deformation, resist aggressive food types, protect the metal of the cans, and preserve the food, maintaining its quality and taste [1].

Since the 1950s, major types of internal can coating have been made from synthetic polymers known as epoxy-based resins, which contain among their components bisphenol A (BPA) or bisphenol A diglycidyl ether (BADGE). Epoxy resins have achieved wide acceptance for use as protective coatings because of their exceptional properties such as toughness, good adhesion to different metal surfaces, formability, and chemical and heat resistance [2]. However, these coatings can release these compounds as well as oligomers and/or derivates (hydrolysed or chlorinated), which can migrate into the packed food. Chlorinated derivatives of BADGE may be generated during the thermal coating treatment, since BADGE is also used as additive to remove the hydrochloric acid formed in this process. Moreover, hydrolysed derivatives of BADGE can be produced during storage when the coating comes into contact with aqueous and acidic foodstuffs [3,4].

There is no specific European legislation for coatings, but there are some harmonized regulations for specific chemicals that are known to migrate from can coatings, such as BADGE and its derivatives (Commission Regulation EC 1895/2005), which establish specific migration limits (SML) to ensure the safety of these materials used for packaging. Recently, Regulation (EU) No 2018/213 was published, reducing the specific migration limit of BPA to 0.05 mg/kg food from plastic materials, but also applicable to varnishes and coatings. Its use, however, is not allowed in articles or materials intended for food contact for small children [4,5,6].

Despite the uncertainties about the adverse effects of BPA, there have been numerous investigations into its properties as endocrine disruptive chemicals, such as reproductive and developmental effects, as well as neurological, immunomodulatory, cardiovascular and metabolic effects [1,7,8,9,10]. It is for this reason that other analogues of the bisphenol family have gradually been developed to partially replace BPA in the manufacturing of these resins and plastics. There are very few data available regarding the safety of these analogues, which consist of two phenolic rings joined by a carbon bridge (Table 1). However, as a consequence of the chemical structure, their possible ability to produce similar adverse effects cannot be excluded [11].

Usually, food industries purchase packaging material already coated with these resins, having no knowledge of the exact composition of these coatings [12]. Can coatings can release complex chemical mixtures into food, but most studies have been focused on the determination of specific compounds such as BPA, BADGE and their derivatives. However, the total migrants from cans may also contain oligomers, prepolymers, catalysts, reaction accelerators, epoxidized edible oils, esters, waxes, lubricants, metals, etc. In addition to intentionally added substances, unintentionally added substances (NIAS) such as impurities, reaction by-products and degradation products generally constitute a part of the migrant [1,13]. Therefore, it is important to develop methods for identifying these migrant compounds, since they could migrate into the food and represent a risk for consumer health. Mass spectrometry coupled to gas chromatography (GC-MS) for volatile and semi-volatile compounds or liquid chromatography (LC-MS) for non-volatile compounds seems like a useful and powerful technology for carrying out these analyses [14].

In the present work, a GC-MS screening for the identification of potential migrants in the can extracts was carried out. Then, in a second step, to assess the possible impact on the health related to bisphenols exposure, a targeted analysis for the simultaneous determination of a series of bisphenols and BADGEs was also performed by LC-MS/MS.

## 2. Materials and Methods

### 2.1. Reagents and Standards

Acetonitrile (ACN) HPLC grade and LC-MS grade and methanol (MeOH) LC-MS grade were provided from Merck (Darmstadt, Germany). Ultrapure water (type I) was obtained from an Autwomatic Plus purification system (Wasserlab, Navarra, Spain).

Analytical standards of 2,6-di-tert-butyl-1,4-benzoquinone 98% (CAS 719-22-2); butylated hydroxytoluene ≥99% (CAS 128-37-0); 2,4-di-tert-butylphenol 99% (CAS 96-76-4); benzophenone 99% (CAS 119-61-9); nonylphenol, technical mixture (CAS 84852-15-3); diisobutyl phthalate 99% (CAS 84-69-5); methyl hexadecanoate 97% (CAS 112-39-0); dibutyl phthalate 99% (CAS 84-74-2); benzoguanamine 97% (CAS 91-76-9); dicyclohexyl phthalate 99% (CAS 84-61-7); bis(2-ethylhexyl) terephthalate ≥96% (CAS 6422-86-2); glycerol trioctanoate ≥99% (CAS 538-23-8) and squalene ≥98% (CAS 111-02-4) were purchased from Sigma-Aldrich (Schnelldorf, Germany); while analytical standards of acetyl tributyl citrate 99% (CAS 77-90-7); bis(2-ethylhexyl) adipate ≥99% (CAS 103-23-1); and bis(2-ethylhexyl) phthalate 99% (CAS 117-81-7) were obtained from Fluka (Steinheim, Germany).

Bisphenol standards: bisphenol A (BPA) ≥99% (CAS 80-05-7) was obtained from Aldrich-Chemie (Steinheim, Germany); bisphenol B (BPB) ≥98% (CAS 77-40-7); bisphenol C (BPC) ≥99% (CAS 79-97-0); bisphenol E (BPE) ≥98% (CAS 2081-08-5); bisphenol F (BPF) ≥98% (CAS 620-92-8); bisphenol G (BPG) ≥98% (CAS 127-54-8); bisphenol A diglycidyl ether (BADGE) ≥95% (CAS 1675-54-3); bisphenol A (3-chloro-2-hydroxypropyl) (2,3-dihydroxypropyl) ether (BADGE.H_2_O.HCl) ≥95% (CAS 227947-06-0); bisphenol A (3-chloro-2-hydroxypropyl) glycidyl ether (BADGE.HCl) ≥90% (CAS 13836-48-1); and bisphenol A (2,3-dihydroxypropyl) glycidyl ether(BADGE.H_2_O) ≥95% (CAS 76002-91-0) were purchased from Sigma-Aldrich (Schnelldorf, Germany); bisphenol A bis(2,3-dihydroxypropyl) ether (BADGE.2H_2_O) ≥97% (CAS 5581-32-8); and bisphenol A bis(3-chloro-2-hydroxypropyl) ether (BADGE.2HCl) ≥99% (CAS 4809-35-2) were obtained from Fluka (Steinheim, Germany); cyclo-di-BADGE (CYDBADGE) 99.5% (CAS 20583-87-3) was obtained from Chiron AS (Trondheim, Norway). The chemical structures and the molecular weight of the selected compounds are given in Table 1.

The stock standard solutions of each bisphenol and BADGEs were individually prepared in acetonitrile and the mix working solutions were made by dilution in 45% acetonitrile (in water). The solutions were stored at 4 °C and brought to room temperature prior to analysis.

### 2.2. Samples and Extraction Procedure

A total of twelve cans covering several types of foods, including fish, seafood, vegetables and fruit, were purchased in a local supermarket in Santiago de Compostela (Spain) and analysed in order to identify potential migrants. Samples were stored at room temperature until analysis. An overview of all of the can samples analysed in the study with their characteristics is presented in Table 2.

The thickness of the lid, the base and the lateral of each can were determined using an Electronic Digital Outside Micrometer (Mitutoyo, Japan). The reported results are an average of three experimental measurements.

To extract the migrants, the cans were opened, emptied and washed with warm water before extraction. The whole can was extracted, a known surface of the packaging was put in contact with a volume of acetonitrile (surface/volume ratio included in Table 2) for 24 h in an oven at 70 °C. The can was covered with aluminium foil to avoid evaporation losses. Then, an aliquot of the extracts (10 mL) was evaporated down to 1 mL by a stream of nitrogen (RapidVap Vertex Evaporator, Labconco, Kansas City, MO, USA) and filtered through a PTFE 0.45 µm filter to be analysed by GC-MS, while another aliquot was diluted to half with water type I and filtered through a PTFE 0.22 µm filter for LC-MS/MS analysis.

### 2.3. Equipment

#### 2.3.1. Fourier Transform Infrared Spectroscopy (FTIR)

To verify the type of coating, infrared spectra were acquired using an ATR (attenuated total reflectance)-FTIR spectrometer (ATR-PRO ONE, FTIR 4700, Jasco, Tokyo, Japan) equipped with a diamond optical crystal. The analysis was done on both sample surfaces (internal and external side), taking into account the base, the lateral and the seam (if it is present) by covering the entire crystal surface using the clamp pressure set to its maximum value to obtain the optimal absorbance. ATR-FTIR spectrometer was controlled by the software Spectra ManagerTM version 2 in the region from 4000 to 650 cm^−1^. The spectra identification was performed by using KnowItAll 17.4.135.B software to compare the sample spectra obtained with the commercial database (IR Spectral Libraries of Polymers & Related Compounds from Bio-Rad Laboratories, Inc. Philadelphia, PA, USA).

#### 2.3.2. GC-MS—Screening Analysis

A Thermo Scientific Trace 1300 Series gas chromatograph with a Trace ISQ LT mass spectrometer detector and an AI 1310 autosampler injector was used to carry out the GC analysis (Thermo Fischer Scientific, San José, CA, USA). The chromatographic conditions were as follows: a ZB-5MS 5% phenyl, 95% dimethylpolysiloxane (30 m × 0.25 mm × 0.25 µm) column from Phenomenex® (Torrance, CA, USA) was used; the injection port temperature was set at 300 °C. The injection mode was splitless, and the injection volume was 1.0 µL. Helium (from Praxair, Madrid, Spain) was used as carrier gas at a constant flow rate of 1 mL/min. The transfer line and source temperature were set at 300 °C. The oven temperature was initially set at 40 °C for 2 min, then increased at a rate of 9 °C/min until 300 °C and held at 300 °C for 3 min. The chromatograms were acquired in full scan mode over m/z range of 35–500. The mass spectra were obtained with a mass-selective detector under electron impact ionization mode at a voltage of 70 eV. For data acquisition and processing, Xcalibur 3.0.63.3 software was used. Mass spectra libraries NIST/EPA/NIH 11 (version 2.0) and Wiley RegistryTM 8th edition were used for identification purposes.

#### 2.3.3. LC-MS/MS—Targeted Analysis

The UPLC-MS/MS system comprised an Accela autosampler, an Accela 1250 pump fitted with a degasser, and a column thermostatting system coupled to a triple quadrupole mass spectrometer TSQ Quantum Access max, controlled by Xcalibur 2.1.0 software (Thermo Fisher Scientific, San José, CA, USA).

The chromatographic separation was achieved on a reversed-phase column Phenosphere 80A ODS (150 mm × 3.2 mm internal diameter, 3 µm particle size) with a pre-column from Phenomenex® (Torrance, CA, USA). Mobile phases were MeOH:ACN (50:50, *v*/*v*) and water. A gradient elution method was applied: for 2 min, the mobile phase was 55% water and 45% MeOH:ACN, and then the concentration of MeOH-ACN was gradually increased, reaching 75% at minute 16, followed by another gradient to 100% MeOH-ACN at minute 23, and finally this composition was held constant until minute 28. The column was kept at 30 °C and the autosampler was maintained at ambient temperature. The flow rate remained constant at 0.5 mL/min, and the injection volume was 10 µL.

The mass spectrometer was operated in positive and negative atmospheric pressure chemical ionisation (APCI) mode for the identification of the compounds. The operating conditions were: nitrogen (from Praxair) which was used as the sheath gas at a pressure of 35 psi, and as auxiliary gas (pressure 10 arbitrary units), argon was used as the collision gas at a pressure of 1.5 mTorr, the vaporizer temperature and capillary temperature were at 400 and 350 °C, respectively. When data were acquired in selected reaction monitoring (SRM) mode, two transitions of each compound were chosen for identification purposes, and the corresponding collision energy was optimized for maximum intensity. MS/MS conditions for bisphenols and BADGEs are given in Table 3.

## 3. Results and Discussion

### 3.1. FTIR Analysis

**Figure 1:**
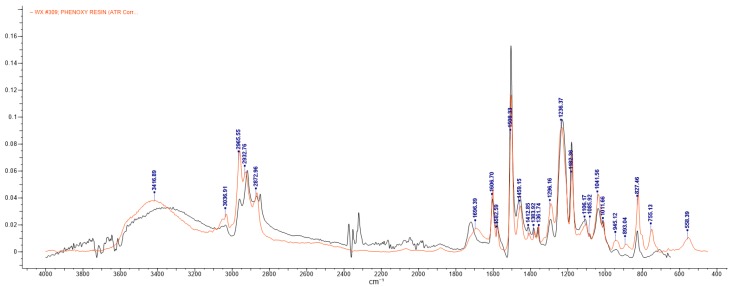
IR spectrum of the internal side of the base in sample ES (**dark line**) compared to the first entry of the IR Spectral Libraries (**red line**).

The FTIR results confirmed that most of the samples examined in this study were coated with epoxy-phenolic resins, as shown in Table 2. The most common epoxy-based coatings are synthesized from bisphenol A and epichlorohydrin forming epoxy resins of bisphenol A diglycidyl ether (BADGE). Many different mixtures within the epoxy coatings have been developed, with the epoxy-phenolic coatings being the most important subgroup. Phenolics are common crosslinkers in epoxide resins and increase their resistance against corrosion and sulphide stains [1]. Figure 1 includes the IR spectrum of the internal side of the base of sample ES, overlaid with the first entry of the IR Spectral Libraries (a phenoxy resin).

However, can manufacturers and food industries have begun to innovate and develop alternatives to replace food contact materials based on BPA epoxy resins as a consequence of reported toxic effects, public discussions, and recent regulatory decisions. Acrylic and polyester coatings are currently in use as first-generation alternatives to epoxy-based coatings and, more recently, polyolefin and non-BPA epoxy coatings have been developed. Acrylic resins have a clean appearance and display corrosion and sulphide stain resistance. Polyester resins, resulting from a condensation reaction between polycarboxylic acids and polyols, have the advantage of being easy to handle during the manufacturing process and adhere well to the metal surface. Isophthalic acid (IPA) and terephthalic acid (TPA) are the main carboxylic acids used in polyester coatings. However, they have the disadvantage of not being stable under acidic conditions, so they cannot be used for acidic food types. Alternatively, polyethylene terephthalate (PET) coatings are used on the inner side and sometimes also on the outside surface of the cans [1]. 

In some cases, it has been observed that the inner lateral sheet had no internal coating. This is common when fruit is packaged (e.g., peach), because tin is more easily oxidized than the food, thus preventing darkening and taste changes caused by the oxidation of the fruit [1]. Some samples had an internal coating known as “side seam stripe” that is applied to welded or cemented seams to protect exposed metal [15]. This coating was scraped and analysed in the FTIR, resulting in PET in all cases. Two of the samples (AN and AR) presented an easy opening system as a lid, the composition of which turned out to be polypropylene on the inner side.

### 3.2. GC-MS Screening

There is very little literature on non-targeted analysis in this type of food packaging. In this study, an approach was carried out to find out what potential migrants could be present in food packaging by analysing samples that were already in contact with the food, since the material was not available prior to contact. Therefore, it should be taken into account that migration could take place in both directions, from the packaging to the food and from the food to the packaging.

Based on our laboratory experience and previous studies, acetonitrile was selected as the most appropriate solvent for carrying out the extraction of the unreacted compounds remaining in the coating. A total of forty-five volatile and semi-volatile compounds were identified in the extracts of the analysed cans (Table 4). Sixteen compounds were positively confirmed by injection of the respective standard comparing the retention times and their respective mass spectra, and the rest of the peaks were tentatively identified by comparison of the mass spectra with the library entries. Only compounds with appropriate direct matching factors (SI) and reverse search matching (RSI) are included in Table 4.

Modern coatings are complex formulas that contains different additives to enhance the properties of the resin, for example, agents to increase surface slipping as well as abrasion and scratch resistance of can coatings, anti-foaming agents, adhesives, scavengers for hydrochloric acids, and pigments among others [1].

Different plasticizers, such as tributyl citrate, acetyl tributyl citrate (ATBC), butyl stearate, and bis(2-ethylhexyl) sebacate, bis(2-ethylhexyl) adipate (DEHA), bis(2-ethylhexyl) fumarate (DEHF), and phthalates were identified in the extracts. Tributyl citrate, detected only in the sample TO1, is used as a plasticizer in inks, adhesives and coatings [16]. ATBC is the most widely used phthalate substitute plasticizer, and was detected in the samples ES, MA and MZ [17]. Butyl stearate, detected in the samples AA, AH and ME, is another type of plasticizer commonly used with low toxicity [18]. DEHA is frequently used as a plasticizer in flexible PVC films, as well as in coatings, printing inks and adhesives [16]. This compound was detected in three samples (AL, AR and ES). Sebacates, such as bis(2-ethylhexyl) sebacate detected in MA, are also used as plasticizers, providing excellent low-temperature flexibility to elastomers [19]. DEHF is a plasticizer and a forming agent for polyester resins, and was found in the sample AA [20]. However, phthalate esters are undoubtedly the most common group of plasticizers present in food packaging. Their control is important, since numerous studies have identified them as endocrine disruptors and related them with harsh effects on the human reproductive system [21]. Among the phthalates, diisobutyl phthalate (DIBP) was detected in all of the samples, while dibutyl phthalate (DBP) was only detected in the sample ES. DIBP is a plasticizer commonly associated with printing inks and it has been also reported as NIAS in plastic films [22]. Bis(2-ethylhexyl) phthalate (DEHP) was detected in all of the samples except AL and ME. Bis(2-ethylhexyl) terephthalate (DEHT), a structural isomer of DEHP, used as an alternative plasticizer was detected in the sample TO2 [23]. Dicyclohexyl phthalate (DCHP) is another phthalate plasticizer detected only in the sample AA. 1-hexanol-2-ethyl, detected in the samples AA and ME, is a volatile compound described as a product formed by thermal decomposition or hydrolysis of plasticizers such as DEHP or DEHA [24].

Butylated hydroxytoluene (BHT), detected in the sample AH, is a synthetic antioxidant commonly used in the food industry as a stabilizer for polyolefins in polyurethane adhesives and it is also employed as a food additive. Its degradation product 2,6-di-tert-butyl-4-methylene-2,5-cyclohexadienone was detected in the sample AH and TO1 [14].

Several degradation products formed from antioxidants used as additives were identified in the can extracts, for example, 1,3-di-tert-butylbenzene and 2,4-di-tert-butylphenol degradation products from antioxidants Irgafos 168 or Irganox 1076 [25] were detected in all samples; 2,6-di-tert-butyl-1,4-benzoquinone degradation products from antioxidants Irgafos 168 and Irganox 1010, frequently detected as NIAS in migration studies [22], was only detected in the sample AA; and 7,9-di-tert-butyl-1-oxaspiro(4,5)deca-6,9-diene-2,8-dione, a degradation product of Irganox 1010, was detected in all the samples [14,26]. Particular attention should be paid to these degradation products, since according to some investigations, they could represent a risk for the health of consumers [14].

The photoinitiators benzophenone, detected in AN and MA extracts, and 4-phenylbenzophenone, detected in AN, are employed, among other applications, for printing inks cured by UV radiation applied to food contact materials, for coatings, adhesives, etc. [17,27,28]. They are not chemically bound to the polymeric structure, and consequently, they can easily migrate into food or beverage [29].

Some compounds that are related to the production of epoxy resins were identified in the extracts, for example, 4-tert-butylbenzoic was present in the sample AL [30]; nonylphenol, a catalyst in the cure of epoxy resins [31] or a starting material for the synthesis of nonylphenol ethoxylates (monomer for phenolic resins) [32], was detected in the sample SR; and benzoguanamine, which is used as cross-linker in, e.g., epoxy- and PVC-based coatings, was detected in three samples (AN, AR and TO1) [1,13].

Several lubricants were found in the samples, such as palmitic, stearic acid [33], ethyl palmitate [16], butyl palmitate [34], oleic acid, and glycerol tricaprylate [27].

Some compounds used in solvents for lacquers and adhesives were identified. Isophorone (3,3,5-trimethyl-2-cyclohexen-1-one) is a constituent in solvent mixtures for finishes and stoving lacquers and was detected in two samples (TO1 and TO2) [35]. Phenol, 2-(1-phenylethyl), detected in sample AA, has been reported in acrylic adhesives [36]. Tricyclodecanedimethanol, detected in AN, is used as a polyol in some of the coatings tested for adhesion [37]. Dehydroabietal, a compound detected in hotmelt adhesives, was present in sample TO2 [38]. Methyl dehydroabietate is a component of varnishes and printing inks [34] used as a tackifier for the enhancement of adhesive performance [26]. This compound was identified in five samples (AN, MA, SR, TO1, TO2). Dehydroabietic acid, detected in the extract of sample AL, is used as solvent for printing inks and preservatives [39]. Methyl abietate is a compound used in lacquers, varnishes and coatings, and was found in sample SR [40].

Other compounds identified were methyl hexadecanoate and methyl oleate, which are used as an intermediate for detergents, emulsifiers, stabilizers, resins, lubricants, plasticizers, and defoamer in food contact coatings [17,34]. Acetophenone was only detected in sample AN; this substance could be produced during the heat degradation of PET [41]. Squalene, an ethylenic-unsaturated hydrocarbon, was detected in all of the samples analysed. This compound has oxygen-scavenging capacity to extend the shelf life of oxygen sensitive products [27]. 2-ethylhexyl diphenyl phosphate is a rubber accelerator, plasticizer, lubricant, and fire retardant, and was found in sample ES [34].

### 3.3. Targeted Analysis

As the FTIR results show, most of the samples examined in this study were coated with epoxy-phenolic resins, so an analytical method based on LC-MS/MS was designed to simultaneously identify bisphenols and BADGEs in the packaging extracts. The analysis was performed under the analytical conditions described above. The positive and negative atmospheric pressure chemical ionisation (APCI) technique was selected because of its better suitability for the majority of these compounds. The mixture of methanol and acetonitrile as mobile phase made it possible to obtain optimal sensitivity in the determination of these compounds.

The mass spectrometry conditions were optimized by direct infusion of 10 ng/µL of each standard solution into the APCI source by use of a built-in syringe pump integrated in the TSQ instrument, mixing it with the mobile phase used for the initial conditions. The two most intense transitions were selected for identification (Table 3). For cyclo-di-BADGE, the most intense precursor ion was the protonated molecular ion [M+H]^+^; while for BPA, BPB, BPC, BPE, BPF, BPG and BADGE.2H_2_O, it was the deprotonated molecule [M-H]^-^. In some cases, adducts are formed with the mobile phase, such as BADGE, BADGE.H_2_O and BADGE.HCl, whose most abundant ions corresponds to these analytes with a molecule of acetonitrile ([M+CH_3_CNH]^+^). The case of BADGE.2HCl is more difficult to explain; it could be explained if the molecule loses its chloride anion and the hydroxyl groups of the chlorohydrin act as nucleophilic reagents, giving rise to a conjugated acid from which the epoxide arises by the elimination of a proton. Thereby, the formed molecule would act as BADGE, forming an adduct with a molecule of acetonitrile (m/z 382), which coincides with the information reported by other authors [42,43]. For BADGE.H_2_O.HCl, the same process as described for BADGE.2HCl takes place at the chlorinated end of the molecule, until the formation of the epoxy ring leading to a molecule of BADGE.H_2_O, which in negative ionization mode the most intense precursor ion observed (m/z 283) comes from the in-source α-cleavage of the ether bond [M-H-C_3_H_6_O_2_]^−^ [44].

**Figure 2:**
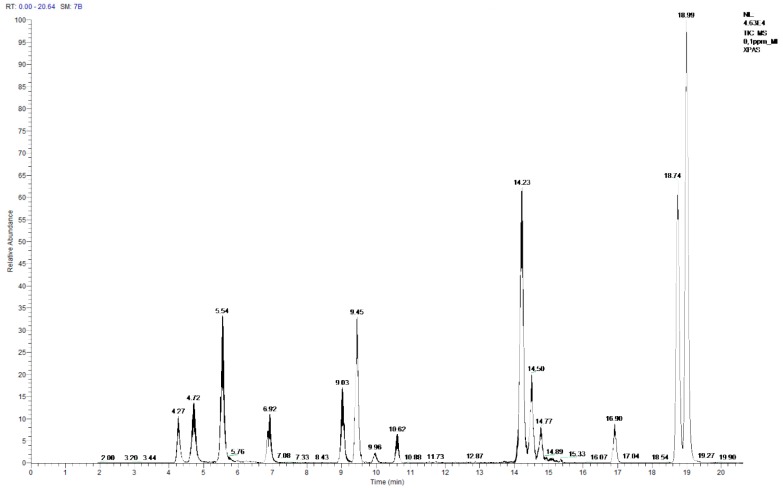
LC-MS/MS chromatogram of a 0.1 mg/L mix working solution.

The sensitivity of the developed method was evaluated by means of limits of detection (LOD), which were estimated as the lowest concentration that provided a signal-to-noise ratio (S/N) higher than three for both transitions. The method showed an excellent sensitivity with LODs of 0.5 µg/L for cyclo-di-BADGE; 1 µg/L for BPE, BPG, and BADGE; 5 µg/L for BPF, BPA, BPB, BPC, BADGE.2H_2_O, BADGE.H_2_O and BADGE.HCl; and 0.5 mg/L for BADGE.2HCl and BADGE.H_2_O.HCl. A LC-MS/MS chromatogram corresponding to a mix working solution of 0.1 mg/L with all the selected compounds is presented in Figure 2. Under these conditions, it was possible to separate the two diastereomers (cis- and trans-) of the cyclo-di-BADGE. Together with each batch of samples, several sample blanks were analysed to evaluate possible background contamination.

Table 5 presents a summary of the bisphenols and BADGEs identified in the extracts of the analysed cans by LC-MS/MS. Among all the bisphenols analysed, only bisphenol A was detected in four samples (AN, ES, ME and TO2). Moreover, BADGE was detected in ten samples (AH, AL, AN, ES, MA, ME, MZ, SR, TO1, TO2). BADGE is found to be unstable in aqueous-based food simulants because it can be hydrolysed, so hydrolysis derivatives (BADGE.H_2_O and BADGE.2H_2_O) may be the best markers for BADGE exposure [45]. These hydrolysis derivatives were detected in all samples, except in sample AR. Regarding the chlorohydroxy derivatives of BADGE, BADGE.HCl was detected in four samples (AH, AL, AN and TO2), BADGE.2HCl was not detected in any samples, and BADGE.H_2_O.HCl was detected in three samples (AL, AN and ME). Cyclo-di-BADGE was detected in all the samples analysed. The most inert sample turned out to be sample AR, where only a small peak corresponding to cyclo-di-BADGE was detected.

As can be observed from the obtained data, BADGEs and BPA were detected in the extraction of the internal side of the can although epoxy resins would not have been identified on this side. This migration was able to take place due to possible set-off phenomena during the manufacturing process and storage of packaging materials in the industry [46]. After applying the coatings, the sheets are stored stacked until they are transported to the manufacturing process. During this time, both sides of the material are in close contact, and the high pressure generated causes the transference of compounds from the outer to the inner face, which can subsequently migrate to the packaged food.

## 4. Conclusions

Two tools were presented for the investigation of potential migrants from coatings. The GC-MS analytical method made it possible, through screening, to identify potential volatile and semi-volatile migrant compounds present in polymeric coatings of food cans. The developed method by LC-MS/MS turned out to be an excellent analytical tool with low detection levels for the positive confirmation of the presence of bisphenols and BADGEs in the can extracts. The results obtained in real food can samples showed that chemicals from polymeric coatings are extractable and should be considered as potential migrants reaching the food that could represent a risk for the consumers′ health. Currently, there is no specific legislation at the European level for this type of FCM. The use of solvent extract is useful to identify compounds that could migrate from containers, but in order to estimate real exposure, analysis of the canned food or migration testing using simulants representing real conditions of use are needed. It would be interesting to know the effect of set-off phenomena in this type of packaging material for food.

## Figures and Tables

**Table 1:** Chemical structures and molecular weight of the selected compounds.

Compound	IUPAC Name	Chemical Structure	Formula	CAS N°	Molecular Weight (g/mol)
BPA	2,2-Bis(4-hydroxyphenyl)propane	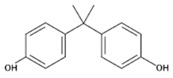	C_15_H_16_O_2_	80-05-7	228.29
BPB	2,2-Bis(4-hydroxyphenyl)butane	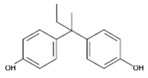	C_16_H_18_O_2_	77-40-7	242.31
BPC	2,2-Bis(4-hydroxy-3-methylphenyl)propane	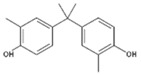	C_17_H_20_O_2_	79-97-0	256.34
BPE	1,1-Bis(4-hydroxyphenyl)ethane	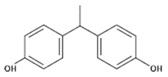	C_14_H_14_O_2_	2081-08-5	214.26
BPF	4.4´-Methylenediphenol	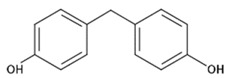	C_13_H_12_O_2_	620-92-8	200.23
BPG	2,2-Bis(4-hydroxy-3-isopropylphenyl)propane	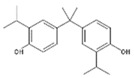	C_21_H_28_O_2_	127-54-8	312.45
BADGE	2,2-Bis(4-hydroxyphenyl)propane bis(2,3-epoxypropyl)ether	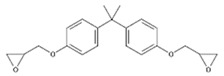	C_21_H_24_O_4_	1675-54-3	340.41
BADGE.H_2_O	3-(4-{2-[4-(2-Oxiranylmethoxy)phenyl]-2-propanyl}phenoxy)-1,2-propanediol	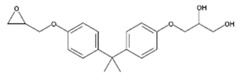	C_21_H_26_O_5_	76002-91-0	358.43
BADGE.2H_2_O	3-(4-{2-[4-(2-Oxiranylmethoxy)phenyl]-2-propanyl}phenoxy)-1,2-propanediol	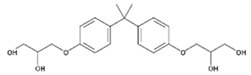	C_21_H_28_O_6_	5581-32-8	376.44
BADGE.HCl	1-Chloro-3-(4-{2-[4-(2-oxiranylmethoxy)phenyl]-2-propanyl}phenoxy)-2-propanol	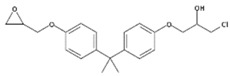	C_21_H_25_ClO_4_	13836-48-1	376.87
BADGE.2HCl	1,1‘-[2,2-Propanediylbis(4,1-phenyleneoxy)]bis(3-chloro-2-propanol)	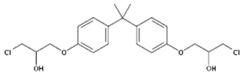	C_21_H_26_C_l2_O4	4809-35-2	413.33
BADGE.H_2_O.HCl	3-(4-{2-[4-(3-Chloro-2-hydroxypropoxy)phenyl]-2-propanyl}phenoxy)-1,2-propanediol	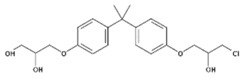	C_21_H_27_ClO_5_	227947-06-0	394.89
CYDBADGE	2,2,10,10-tetramethyl-4,8,12,16-tetraoxa-1,3,9,11(1,4)-tetrabenzenacyclohexadecaphane-6,14-diol	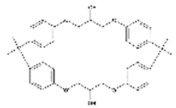	C_36_H_40_O_6_	20583-87-3	568.71

**Table 2:** Details of the samples included in the study.

Coding	Surface/Volume Ratio (dm^2^/mL)*	Thickness (µm)	Type of Material	
			Internal	External
**ES**	0.01	*Lid*: 205.0 *Lateral*: 167.5 *Base*: 177.0	*Lid*: Polyvinyl chloride and thermoplastic urethane *Lateral*: Phenoxy resin *Base*: Phenoxy resin *Seam*: PS (PET)	*Lid*: Phenoxy resin *Lateral*: Phenoxy resin *Base*: Phenoxy resin
**TO1**	0.01	*Lid*: 220.5 *Lateral*: 153.5 *Base*: 188.0	*Lid*: PS (PET) *Lateral*: - *Base*: Poly(1,4-cyclohexanedimethyleneterephthalate) *Seam*: PS (PET)	*Lid*: Phenoxy resin *Lateral*: Polyethylenes *Base*: Phenoxy resin
**TO2**	0.013	*Lid*: 215.5 *Lateral*: 161.0 *Base*: 186.0	*Lid*: PS (PET) *Lateral*: PS (PET) *Base*: Poly(1,4-cyclohexanedimethyleneterephthalate) *Seam*: PS (PET)	*Lid*: Phenoxy resin *Lateral*: Phenoxy resin *Base*: Phenoxy resin
**AH**	0.009	*Lid*: 188.5 *Lateral*: 161.0 *Base*: 167.5	*Lid*: Epoxy base *Lateral*: - *Base*: Solid epoxy resin produced from BPA and epichlorohydrin *Seam*: PS (PET)	*Lid*: Phenoxy resin *Lateral*: PS urethane foam *Base*: Phenoxy resin
**AL**	0.015	*Lid*: 233.0 *Base*: 155.5	*Lid*: Epoxy base *Base*: Epoxy base	*Lid*: Phenoxy resin *Base*: Phenoxy resin
**AA**	0.015	*Lid*: 188.5 *Base*: 150.5	*Lid*: terephthalic acid PS with aliphatic diol *Base*: PS (PET)	*Lid*: Solid epoxy resin produced from BPA and epichlorohydrin *Base*: Phenoxy resin
**ME**	0.02	*Lid*: 208.5 *Base*: 158.5	*Lid*: Epoxy base *Base*: Epoxy base	*Lid*: Phenoxy resin *Base*: Solid epoxy resin produced from BPA and epichlorohydrin
**SR**	0.02	*Lid*: 196.0 *Base*: 155.0	*Lid*: Unsaturated isophthalic PS resin *Base*: Poly(hexamethylene isophthalate)	*Lid*: Phenoxy resin *Base*: Solid epoxy resin produced from BPA and epichlorohydrin
**AN**	0.013	*Lid*: 114.0 *Lateral*: 149.5 *Base*: 240.5	*Lid*: PP-graft-maleic anhydride *Lateral*: Phenoxy resin *Base*: Phenoxy resin *Seam*: PS (PET)	*Lid*: PS-based thermoplastic urethane elastomer *Latera*l: PUR/PVC compound *Base*: Phenoxy resin
**AR**	0.012	*Lid*: 119.5 *Lateral*: 167.5 *Base*: 173.5	*Lid*: PP-graft-maleic anhydride *Lateral*: - *Base*: PS (PET)	*Lid*: PS-based thermoplastic urethane elastomer *Lateral*: PS urethane acrylate *Base*: Phenoxy resin
**MA**	0.01	*Lid*: 218.0 *Lateral*: 140.0 *Base*: 165.5	*Lid*: Epoxy base *Lateral*: - *Base*: Phenoxy resin *Seam*: PS (PET)	*Lid*: Phenoxy resin *Lateral*: - *Base*: Phenoxy resin
**MZ**	0.01	*Lid*: 179.0 *Lateral*: 138.5 *Base*: 162.0	*Lid*: Poly(diallyl isophthalate) *Lateral*: PS (PET) *Base*: Poly(1,4-cyclohexanedimethyleneterephthalate) *Seam*: PS (PET)	*Lid*: Solid epoxy resin produced from BPA and epichlorohydrin *Lateral*: - *Base*: Phenoxy resin

PUR: Polyurethane; PP: Polypropylene; PET: Polyethylene terephthalate; PS: Polyester; -: not coating; *: for extraction purposes.

**Table 3:** MS/MS conditions for the selected bisphenols and BADGEs with their retention times.

Compound	Retention Time (min)	APCI	Parention	Production	Collision Gas Energy (V)
BPF	4.27	-	198.9	93.0	24
				105.0	23
BADGE.2H_2_O	4.72	-	374.8	226.8	28
				300.6	16
BPE	5.54	-	212.9	196.8	33
				197.8	20
BPA	6.92	-	226.9	133.0	28
				211.8	20
BPB	9.03	-	240.9	210.7	31
				211.8	20
BADGE.H_2_O	9.45	+	399.9	106.9	45
				134.8	26
BADGE.H_2_O.HCl	9.96	-	283.0	211.0	30
				226.0	21
BPC	10.62	-	254.9	146.9	33
				239.8	21
BADGE	14.23	+	381.9	134.9	31
				190.8	25
BADGE.HCl	14.50	+	417.9	106.9	43
				134.9	28
BADGE.2HCl	14.77	+	382.2	191.1	16
				135.2	26
BPG	16.90	-	311.0	174.9	33
				294.9	37
CYDBADGE	18.74, 18.99	+	569.0	134.8	29
				106.9	39

**Table 4:** Compounds identified in the extracts of the analysed cans by GC-MS.

TR (min)	CAS N°	Compound	*m/z*	SI	RSI	Sample											
						AA	AH	AL	AN	AR	ES	MA	ME	MZ	SR	TO1	TO2
8.50	104-76-7	1-hexanol-2-ethyl	57, 41	893	953	X							X				
10.19	78-59-1	Isophorone	82, 138	935	936											X	X
12.26	1014-60-4	1,3-di-tert-butylbenzene	57, 175	814	837	X	X	X	X	X	X	X	X	X	X	X	X
14.02	98-86-2	Acetophenone	105, 120	706	946				X								
14.19	98-73-7	4-tert-Butylbenzoic acid	135, 163	811	876			X									
15.50	719-22-2	2,6-Di-tert-butyl-1,4-benzoquinone*	177, 220	709	759	X											
15.63	2607-52-5	2,6-Di-tert-butyl-4-methylene-2,5-cyclohexadienone	161, 203	866	889		X									X	
16.06	128-37-0	Butylated hydroxytoluene*	205, 220	908	920		X										
16.09	96-76-4	2,4-Di-tert-butylphenol *	191, 206	894	924	X	X	X	X	X	X	X	X	X	X	X	X
16.85	143-07-7	Dodecanoic acid	60, 73	823	890							X					
17.84	119-61-9	Benzophenone*	77, 105	895	960				X			X					
18.37	24157-81-1	2,6-Diisopropylnaphthalene	155, 197	724	869				X								
18.51	84852-15-3	Nonylphenol*	121, 163	700	878										X		
18.91	4237-44-9	2-(1-Phenylethyl) phenol	183, 198	807	963	X											
19.66	26896-48-0	Tricyclodecanedimethanol	79, 91	824	851				X								
20.52	84-69-5	DIBP*	149, 223	802	888	X	X	X	X	X	X	X	X	X	X	X	X
21.04	82304-66-3	7,9-Di-tert-butyl-1-oxaspiro[4.5]deca-6,9-diene-2,8-dione	175, 205	802	870	X	X	X	X	X	X	X	X	X	X	X	X
21.24	112-39-0	Methyl hexadecanoate*	74, 87	733	827	X	X										
21.59	84-74-2	DBP*	149, 150	718	888						X						
21.65	57-10-3	Palmitic acid	73, 129	893	907	X	X	X	X	X	X	X	X	X	X		X
21.98	628-97-7	Ethyl palmitate	88, 101	798	829		X							X	X	X	X
22.38	91-76-9	Benzoguanamine*	103, 187	916	940				X	X						X	
23.11	112-62-9	Methyl oleate	55, 69	874	894	X	X	X			X				X		
23.50	112-80-1	Oleic acid	73, 129	807	893					X							
23.75	57-11-4	Stearic Acid	43, 73	853	898	X	X	X	X		X	X		X	X		
23.93	77-94-1	Tributyl citrate	129, 185	733	811											X	
23.97	111-06-8	Butyl Palmitate	56, 257	718	751	X											
24.36	141-02-6	Bis(2-ethylhexyl) fumarate (DEHF)	70, 112	834	888	X											
24.56	77-90-7	ATBC*	129, 185	782	891						X	X		X			
24.91	13601-88-2	Dehydroabietal	241, 269	796	919												X
25.51	1235-74-1	Methyl Dehydroabietate	299, 239	739	828				X			X			X	X	X
25.60	17611-16-4	(13β)-Abiet-8-en-18-oic acid	243, 289	734	743			X									
25.89	123-95-5	Butyl stearate	56, 285	800	826	X	X						X				
25.90	103-23-1	DEHA*	112, 129	707	848			X		X	X						
25.99	127-25-3	Methyl Abietate	121, 256	886	925										X		
26.21	1241-94-7	2-Ethylhexyl diphenyl phosphate	251, 362	767	857						X						
26.43	1740-19-8	Dehydroabietic acid	239, 285	712	855			X									
26.66	2128-93-0	4-Phenylbenzophenone	152, 181	700	808				X								
27.06	84-61-7	DCHP*	149, 167	888	910	X											
27.18	117-81-7	DEHP*	149, 167	942	942	X	X		X	X	X	X		X	X	X	X
27.19	791-28-6	Triphenylphosphine oxide	199, 277	831	864			X					X				
28.94	6422-86-2	DEHT*	112, 261	704	858												X
29.36	122-62-3	Bis(2-ethylhexyl) sebacate	112, 185	781	837							X					
29.50	111-02-4	Squalene*	69, 81	949	949	X	X	X	X	X	X	X	X	X	X	X	X
30.43	538-23-8	Glycerol tricaprylate*	57, 127	811	858					X		X					X

*: confirmed with standards.

**Table 5:** Bisphenols and BADGEs identified in the extracts of the analysed cans by LC-MS/MS.

Compound	Samples											
	AA	AH	AL	AN	AR	ES	MA	ME	MZ	SR	TO1	TO2
BPF												
BADGE.2H_2_O	X	X	X	X		X	X	X	X	X	X	X
BPE												
BPA				X		X		X				X
BPB												
BADGE.H_2_O	X	X	X	X		X	X	X	X	X	X	X
BADGE.H_2_O.HCl			X	X				X				
BPC												
BADGE		X	X	X		X	X	X	X	X	X	X
BADGE.HCl		X	X	X								X
BADGE.2HCl												
BPG												
CYDBADGE	X	X	X	X	X	X	X	X	X	X	X	X

## References

[1] Geueke B. (2016). FPF Dossier: Can Coatings.

[2] Alwan R.M., Ali R.A., Hasan H.A., Mohammed A., Ali N.A. (2015). Study of new users of internal coating for Food and beverage cans. Int. J. Chem. Stud..

[3] Gallart-Ayala H., Moyano E., Galceran M.T. (2011). Fast liquid chromatography–tandem mass spectrometry for the analysis of bisphenol A-diglycidyl ether, bisphenol F-diglycidyl ether and their derivatives in canned food and beverages. J. Chromatogr. A.

[4] Paseiro Losada P., Simal Lozano J., Paz Abuin S., López Mahia P., Simal Gándara J. (1993). Kinetics of the hydrolysis of bisphenol diglycidyl ether (BADGE) in water-based food simulants. J. Anal. Chem..

[5] European Commission (2011). Commission Regulation (EU) No. 10/2011, on Plastic Materials and Articles Intended to Come into Contact with Food. Off. J. Eur. Union.

[6] European Commission (2018). Commission Regulation (EU) No. 2018/213, on 12 February 2018 on the Use of Bisphenol A in Varnishes and Coatings Intended to Come into Contact with Food. Off. J. Eur. Union.

[7] Driffield M., Garcia-Lopez M., Christy J., Lloyd A.S., Tarbin J.A., Hough P., Bradley E.L., Oldring P.K.T. (2018). The determination of monomers and oligomers from polyester-based can coatings into foodstuffs over extended storage periods. Food Addit. Contam. Part A.

[8] Paseiro-Cerrato R., MacMahon S., Ridge C.D., Noonan G.O., Begley T.H. (2016). Identification of unknown compounds from polyester cans coatings that may potentially migrate into food or food simulants. J. Chromatogr. A.

[9] Paseiro-Cerrato R., Noonan G.O., Begley T.H. (2016). Evaluation of long-term migration testing from can coatings into food simulants: Polyester coatings. J. Agric. Food Chem..

[10] Lestido Cardama A., Rodríguez Bernaldo de Quirós A., Sendón R. (2017). Analysis of bisphenol A in beverages and food packaging by high-performance liquid chromatography. Food Nutr. J..

[11] Russo G., Barbato F., Grumetto L. (2016). Development and validation of a LC-FD method for the simultaneous determination of eight bisphenols in soft drinks. Food Anal. Methods.

[12] Sendón García R., Paseiro Losada P., Pérez Lamela C. (2003). Determination of compounds from epoxy resins in food simulants by HPLC-fluorescence. Chromatographia.

[13] Paseiro-Cerrato R., DeVries J., Begley T.H. (2017). Evaluation of short-term and long-term migration testing from can coatings into food simulants: Epoxy and acrylic−phenolic coatings. J. Agric. Food Chem..

[14] Galmán Graíño S., Sendón R., López Hernández J., Rodríguez-Bernaldo de Quirós A. (2018). GC-MS Screening Analysis for the Identification of Potential Migrants in Plastic and Paper-Based Candy Wrappers. Polymers.

[15] U. S. Environmental Protection Agency (1998). Preliminary Industry Characterization: Metal Can Manufacturing—Surface Coating.

[16] García Ibarra V., Sendón R., Bustos J., Paseiro Losada P., Rodríguez Bernaldo de Quirós A. (2019). Estimates of dietary exposure of Spanish population to packaging contaminants from cereal based foods contained in plastic materials. Food Chem. Toxicol..

[17] García Ibarra V., Rodríguez Bernaldo de Quirós A., Paseiro Losada P., Sendón R. (2018). Identification of intentionally and non-intentionally added substances in plastic packaging materials and their migration into food products. Anal. Bioanal. Chem..

[18] Lau O.W., Wong S.K. (2000). Contamination in food from packaging material. J. Chromatogr. A.

[19] Rodríguez Bernaldo de Quirós A., Lestido Cardama A., Sendón R., García Ibarra V. (2019). Food Contamination by Packaging: Migration of Chemicals from Food Contact Materials.

[20] Vápenka L., Vavrouš A., Votavová L., Kejlová K., Dobiáš J., Sosnovcová J. (2016). Contaminants in the paper-based food packaging materials used in the Czech Republic. J. Food Nutr. Res..

[21] Lago M.A., Ackerman L.K. (2016). Identification of print-related contaminants in food packaging. Food Addit. Contam. Part A.

[22] Cherta L., Portolés T., Pitarch E., Beltran J., López F.J., Calatayud C., Company B., Hernández F. (2015). Analytical strategy based on the combination of gas chromatography coupled to time-of-flight and hybrid quadrupole time-of-flight mass analyzers for non-target analysis in food packaging. Food Chem..

[23] Lessmann F., Correia-Sá L., Calhau C., Domingues V.F., Weiss T., Brüning T., Koch H.M. (2017). Exposure to the plasticizer di (2-ethylhexyl) terephthalate (DEHTP) in Portuguese children–Urinary metabolite levels and estimated daily intakes. Environ. Int..

[24] Kusch P., Warren V. (2017). The Application of Headspace: Solid-Phase Microextraction (HS-SPME) Coupled with Gas Chromatography/Mass Spectrometry (GC/MS) for the Characterization of Polymers. Gas Chromatography, Analysis, Methods and Practices.

[25] Demertzis P.G., Franz R., Welle F. (1999). The effects of γ-irradiation on compositional changes in plastic packaging films. Packag. Technol. Sci..

[26] Domeño C., Aznar M., Nerín C., Isella F., Fedeli M., Bosetti O. (2017). Safety by design of printed multilayer materials intended for food packaging. Food Addit. Contam. Part A.

[27] García Ibarra V., Rodríguez Bernaldo de Quirós A., Paseiro Losada P., Sendón R. (2019). Non-target analysis of intentionally and non intentionally added substances from plastic packaging materials and their migration into food simulants. Food Packag. Shelf.

[28] Bradley E.L., Stratton J.S., Leak J., Lister L., Castle L. (2013). Printing ink compounds in foods: UK survey results. Food Addit. Contam. Part B.

[29] Simon C., Onghena M., Covaci A., Van Hoeck E., Van Loco J., Vandermarken T., Van Langenhove K., Demaegdt H., Mertens B., Vandermeiren K. (2016). Screening of endocrine activity of compounds migrating from plastic baby bottles using a multi-receptor panel of in vitro bioassays. Toxicol. Vitr..

[30] Rodriguez-Gonzalo E., García-Gómez D., Carabias-Martínez R. (2010). A confirmatory method for the determination of phenolic endocrine disruptors in honey using restricted-access material–liquid chromatography–tandem mass spectrometry. Anal. Bioanal. Chem..

[31] Oscar N., Hector G.A. (2015). Fast Liquid Chromatography-Mass Spectrometry Methods in Food and Environmental Analysis.

[32] Kolossa-Gehring M., Fiddicke U., Leng G., Angerer J., Wolz B. (2017). New human biomonitoring methods for chemicals of concern—The German approach to enhance relevance. Int. J. Hyg. Environ. Health.

[33] Vera P., Canellas E., Nerín C. (2018). Identification of non volatile migrant compounds and NIAS in polypropylene films used as food packaging characterized by UPLC-MS/QTOF. Talanta.

[34] Dupáková Z., Dobiáš J., Votavová L., Klaudisová K., Voldrich M. (2010). Occurrence of extractable ink residuals in packaging materials used in the Czech Republic. Food Addit. Contam..

[35] Skjevrak I., Brede C., Steffensen I.L., Mikalsen A., Alexander J., Fjeldal P., Herikstad H. (2005). Non-targeted multi-component analytical surveillance of plastic food contact materials: Identification of substances not included in EU positive lists and their risk assessment. Food Addit. Contam..

[36] Canellas E., Vera P., Nerín C. (2015). Risk assessment derived from migrants identified in several adhesives commonly used in food contact materials. Food Chem. Toxicol..

[37] Carter W., Lamb K., Jupina M. (2008). Tougher Cycloaliphatic Epoxide Resins. U.S. Patent Application.

[38] Vera P., Aznar M., Mercea P., Nerín C. (2011). Study of hotmelt adhesives used in food packaging multilayer laminates. Evaluation of the main factors affecting migration to food. J. Mater. Chem..

[39] Rani M., Shim W.J., Han G.M., Jang M., Al-Odaini N.A., Song Y.K., Hong S.H. (2015). Qualitative analysis of additives in plastic marine debris and its new products. Arch. Environ. Contam. Toxicol..

[40] Wypych A. (2017). Databook of Plasticizers.

[41] Dutra C., Pezo D., de Alvarenga Freire M.T., Nerín C., Reyes F.G.R. (2011). Determination of volatile organic compounds in recycled polyethylene terephthalate and high-density polyethylene by headspace solid phase microextraction gas chromatography mass spectrometry to evaluate the efficiency of recycling processes. J. Chromatogr. A.

[42] Pardo O., Yusà V., León N., Pastor A. (2006). Determination of bisphenol diglycidyl ether residues in canned foods by pressurized liquid extraction and liquid chromatography–tandem mass spectrometry. J. Chromatogr. A.

[43] Sendón García R., Paseiro Losada P. (2004). Determination of bisphenol A diglycidyl ether and its hydrolysis and chlorohydroxy derivatives by liquid chromatography–mass spectrometry. J. Chromatogr. A.

[44] Gallart-Ayala H., Moyano E., Galceran M.T. (2010). Multiple-stage mass spectrometry analysis of bisphenol A diglycidyl ether, bisphenol F diglycidyl ether and their derivatives. Rapid Commun. Mass Spectrom..

[45] Chang Y., Nguyen C., Paranjpe V.R., Gilliland F., Zhang J.J. (2014). Analysis of bisphenol A diglycidyl ether (BADGE) and its hydrolytic metabolites in biological specimens by high-performance liquid chromatography and tandem mass spectrometry. J. Chromatogr. B.

[46] Clemente I., Aznar M., Nerín C., Bosetti O. (2016). Migration from printing inks in multilayer food packaging materials by GC-MS analysis and pattern recognition with chemometrics. Food Addit. Contam. Part. A.

